# Different fungal signatures in ALD and MAFLD

**DOI:** 10.3389/fmicb.2024.1510507

**Published:** 2024-11-27

**Authors:** Daya Zhang, Qi Wang, Da Li, Chen Chen, Yanting Lv, Shimei Huang, Fan Zeng, Xianfeng Huang, Fengjiao Mao, Feihu Bai

**Affiliations:** ^1^Graduate School, Hainan Medical University, Haikou, China; ^2^Department of Gastroenterology, The Second Affiliated Hospital of Hainan Medical University, Haikou, China; ^3^The Gastroenterology Clinical Medical Center of Hainan Province, Haikou, China

**Keywords:** ALD, MAFLD, fecal samples, mycobiome, microbiome, fungi

## Abstract

**Objective:**

This study investigates the differential impact of fecal fungal microbiota on the pathogenesis of alcohol-associated liver disease (ALD) and metabolic-associated fatty liver disease (MAFLD). We aim to delineate distinct microbial patterns across various stages of each disease.

**Methods:**

We conducted fungal internal transcribed spacer 2 (ITS2) sequencing analysis on fecal samples from 48 ALD patients, 55 MAFLD patients, and 64 healthy controls (HCs).

**Results:**

Distinct fungal microbiota profiles were significantly identified between the ALD and MAFLD patients. In the ALD group, genera such as Trichosporon, Davidiella and Asterotremella along with species like Trichosporon unclassified and Davidiella unclassified were elevated compared to those in the MAFLD group. Conversely, Fungi unclassified, Rhizopus, Periconia, and *Candida albicans* were more prevalent in MAFLD patients. A specific fungal signature comprising Asterotremella_pseudolonga, Malassezia_restricta and Malassezia, was notably effective in differentiating ALD from MAFLD, achieving an area under the curve (AUC) of 0.94. Periconia and Periconia byssoides were more abundant in non-obese MAFLD patients compared to obese MAFLD and HCs. Rhizopus microsporus var. chinensis and var. rhizopodiformis, along with Pleosporales unclassified, were predominantly found in MAFLD patients with moderate to severe hepatic steatosis (HS). The genera Pleosporales_unclassified and the species Candida_albicans were markedly elevated in ALC patients when contrasted with AFL or HCs.

**Conclusion:**

This investigation introduces a novel fungal signature that successfully differentiates between ALD and MAFLD, underscoring Pleosporales unclassified, as biomarkers for disease progression in ALD and MAFLD. The findings also suggest a significant role for Periconia in the progression of non-obese MAFLD.

## 1 Introduction

Alcoholic liver disease (ALD) and Metabolic Associated Fatty Liver Disease (MAFLD) are major contributors to global morbidity and mortality, with their prevalence steadily increasing (Eslam et al., 2020; Huang et al., 2023; Younossi et al., 2023). ALD, a significant chronic liver condition worldwide, progresses through four well-defined stages: alcoholic fatty liver (AFL), alcoholic hepatitis (AH), alcoholic liver fibrosis, and alcoholic cirrhosis (ALC) (Singal et al., 2018). Recent studies have highlighted a notable increase in ALD prevalence in Asia, rising from 3.82% during 2000–2010 to 6.62% in 2011–2020, with particularly high rates of ALC and hepatocellular carcinoma (HCC) at 12.57 and 8.30%, respectively. This trend suggests that ALD may soon become the leading cause of chronic liver disease in the region (Xu et al., 2022). Similarly, MAFLD affects a significant portion of the global adult population, with an alarming prevalence of 29.2% in China alone (Younossi et al., 2019). This disease, characterized by hepatic steatosis (HS) and metabolic associated steatohepatitis (MASH), frequently progresses to severe fibrosis and cirrhosis (Zeybel et al., 2022). Intriguingly, non-obese individuals with MAFLD often experience more severe liver complications than their obese counterparts, underscoring the complex nature of this disease (Iwaki et al., 2021).

The pathogenesis of both ALD and MAFLD involves multifactorial and poorly understood mechanisms, with currently no effective treatments established (Targher et al., 2021; Hyun et al., 2021). Thus, identifying novel therapeutic targets and developing effective treatments are of paramount importance. While the majority of existing research has focused on the role of bacterial microbiota in these liver diseases, studies exploring the impact of fungal microbiota are limited and recent (Hyun et al., 2021; Lang et al., 2020).

This study aims to fill this gap by exploring the alterations in fungal microbiota between ALD and MAFLD patients. By examining these changes across different stages of each disease, we seek to uncover distinct fungal patterns that may influence disease progression and potentially offer new targets for therapeutic intervention.

## 2 Materials and methods

### 2.1 Study design and participant enrollment

This study was conducted in Hainan Province, China, from October 2022 to July 2023. It involved 167 participants: 64 healthy controls (HCs), 48 with ALD, and 55 with MAFLD. Participants were adults aged 18 ~ 65, diagnosed with either ALD or MAFLD according to international consensus guidelines (Eslam et al., 2020; Dunn and Shah, 2016). Diagnosis was further refined using ultrasound assessments by a blinded physician, categorizing HS as mild (diffuse echogenic enhancement) or moderate to severe (bright echogenicities with ultrasound beam attenuation). Subgroups within the MAFLD cohort included 20 with moderate to severe HS (msMAFLD) and 35 with mild HS (miMAFLD), along with 37 non-obese (nobMAFLD) and 18 obese individuals (obMAFLD). For ALD, subgroups of 28 with AFL and 20 with ALC were analyzed. This research received ethical approval from the Institutional Review Board of Hainan Medical University (Approval No. HYLL-2023-453), conforming to the Declaration of Helsinki guidelines. All participants provided written informed consent.

### 2.2 Inclusion and exclusion criteria for MAFLD and ALD

Participants with MAFLD met criteria including varying degrees of HS on ultrasound and at least one of the following: BMI ≥ 23 kg/m^2^, type 2 diabetes (fasting glucose ≥7.0 mmol/L), or metabolic dysfunction. ALD criteria required a history of significant alcohol intake (>40 g/day) with imaging evidence of HS or cirrhosis. Exclusions were applied for viral hepatitis, drug-induced liver disease, severe cardiovascular or renal diseases, recent antibiotics or other gut microbiota-affecting drugs, abnormal diets, and significant recent dietary changes.

Detailed criteria for inclusion and exclusion of HCs are detailed in the Supplementary material.

### 2.3 Assessment of dietary patterns and clinical characteristics

Detailed demographic and clinical data were collected, including dietary habits assessed via a standardized food frequency questionnaire (Sun et al., 2021). Blood samples were obtained after overnight fasting for biochemical analyses conducted at our central laboratory. Variables such as serum K^+^, Na^+^, Cl^−^, Ca^+^, alanine aminotransferase (ALT), aspartate aminotransferase (AST), glutamyl transpeptidase (GGT), alkaline phosphatase (ALP), total bilirubin (TBIL), direct bilirubin (DBIL), indirect bilirubin (IBIL), creatinine (Cr), uric acid (URIC), blood urea nitrogen (BUN), high-density lipoprotein cholesterol (HDL-C), triglycerides (TG), cholesterol (CHOL), low-density lipoprotein cholesterol (LDL-C), fasting plasma glucose (FPG), along with blood cell counts, including neutrophils (Neu), lymphocytes (Lym), monocytes (Mono), red blood cell distribution width (RDW), platelets (PLT), and other standard blood tests, were analyzed. Detailed information is summarized in Supplementary Dataset 1.

### 2.4 Sample collection

Fecal samples were immediately processed into three 100 mg aliquots, snap-frozen in liquid nitrogen, and stored at −80°C until analysis.

### 2.5 Fecal DNA extraction and fungal sequencing

Fecal DNA was extracted using the Fecal Genome DNA Extraction Kit (DP712-02, TIANGEN, China) (Karlsson et al., 2014). DNA quality was quantified with Qubit (Invitrogen, United States), and PCR amplified using ITS1FI2/ITS2 primers. Following pooling, the samples were sequenced on an Illumina NovaSeq 6000 (PE250) by Shanghai Biotree Biotech Co., Ltd. The sequencing primers were excised from demultiplexed raw sequences using cutadapt (v1.9). Paired-end reads were merged via FLASH (v1.2.8). Reads failing to meet quality standards (quality scores <20), those <100 bp in length, and reads with over 5% “N” records were trimmed utilizing the sliding-window algorithm in fqtrim (v0.94). Subsequently, quality filtering was executed to obtain high-quality clean tags using fqtrim. Chimeric sequences were filtered using Vsearch software (v2.3.4), and DADA2 was utilized for denoising and generating amplicon sequence variants (ASVs).

### 2.6 Statistics

Categorical variables were analyzed employing Pearson’s chi-squared test, and outcomes are reported as numbers and percentages, unless specified differently. Not continuous outcome data are detailed as medians with upper and lower quartiles in brackets, unless otherwise indicated. Fungal diversity was assessed using the Shannon and inverse Simpson indices, and *β*-diversity was visualized through principal coordinate analysis (McMurdie and Holmes, 2013; Segata et al., 2011). Data were analyzed using the Kruskal-Wallis and Wilcoxon rank-sum tests, with significance adjusted for multiple comparisons using Holm’s method. Significant fungal taxa differences were identified using Linear Discriminant Analysis Effect Size (LEfSe) (McMurdie and Holmes, 2013; Segata et al., 2011). Statistical tests were two-tailed, with a significance level of *p* ≤ 0.05 indicating statistical significance.

## 3 Results

### 3.1 Study population with ALD and MAFLD

As summarized in Table 1, median BMI was significantly higher in both liver disease cohorts compared to HCs, with MAFLD patients showing a notably higher BMI than ALD patients. Key biochemical parameters such as DBIL, AST, GGT, FPG, and URIC were elevated in liver disease groups compared to HCs and ALD patients exhibited significantly higher levels of these markers than MAFLD patients. Additionally, levels of ALT, TG, and LDL-C were increased, whereas HDL-C was lower across both disease groups vs. HCs, achieving statistical significance. Exceptions included TBIL, TP, ALB, and RBC, which showed a trend toward significance when comparing HCs to the ALD group, and CHOL, which exhibited a similar trend when comparing HCs to the MAFLD group. Furthermore, IBIL levels were notably higher in the ALD group than in the MAFLD group.

**Table 1:** Baseline demographic and laboratory data for the study population.

	Healthy controls (HCs) (*n* = 64)	Alcohol-associated liver disease (ALD) (*n* = 48)	Metabolic associated fatty liver disease (MAFLD) (*n* = 55)	*χ*^2^/*H*	*p*
Age (year)	45.00 (38.25–54.00)	50.00 (40.00–56.00)	51.00 (41.00–54.00)	2.978	0.226
Gender [male], *n* [%]	25 (39.06%)	14 (29.1%)	18 (32.72%)	5.538	0.063
BMI (kg/cm^2^)	22.12 (20.49–23.30)	23.39 (321.62–25.76)	26.09 (23.29–29.29)	43.474	<0.001*
TBIL (μmol/L)	12.05 (9–15.8)	15.25 (11.3–26.3)	11.7 (9.6–13.7)	13.494	0.001*
DBIL (μmol/L)	3.4 (2.7–5.25)	4.75 (3.1–11.625)	3.1 (2.4–4)	18.197	<0.001*
IBIL (μmol/L)	9.25 (7.125–12.7)	10.4 (7.5–14.575)	8.6 (6.8–10.5)	4.542	0.103
ALT (IU/L)	17 (14–23)	30 (21.5–46.75)	26 (19–37)	34.247	<0.001*
AST (IU/L)	20 (17–24)	33.5 (23.5–58.75)	23 (19–27)	39.973	<0.001*
GGT (IU/L)	22 (17–29.75)	97.5 (46–198.25)	29 (20–44)	64.662	<0.001*
TP (g/L)	73.8 (70.275–77.225)	70.15 (64.575–74.8)	74.4 (71.9–77.1)	12.349	0.002*
ALB (g/L)	44.55 (42.625–45.775)	41.55 (35.8–44.95)	44.4 (42.9–45.9)	14.353	0.001*
GLB (g/L)	29.65 (26.8–31.775)	29.1 (26.3–33.275)	30 (28.1–31.8)	1.214	0.545
FPG (mmol/L)	4.96 (4.6575–5.21)	5.785 (5.2075–6.5125)	5.08 (4.77–5.9)	25.503	<0.001*
URIC (μmol/L)	310 (260.5–333.25)	390.5 (342–446)	375 (298–409)	38.890	<0.001*
TG (mmol/L)	0.855 (0.69–1.2025)	1.44 (1.18–2.3125)	1.31 (0.98–2.19)	37.626	<0.001*
CHOL (mmol/L)	4.945 (4.3–5.3325)	5.15 (3.8975–6.5025)	5.14 (4.71–5.89)	7.497	0.024*
HDL-C (mmol/L)	1.61 (1.4525–1.9225)	1.31 (1.0125–1.6075)	1.36 (1.12–1.58)	27.370	<0.001*
LDL-C (mmol/L)	2.82 (2.2425–3.075)	3.205 (2.25–4.21)	3.12 (2.54–3.79)	11.136	0.004*
WBC (10^9^/L)	5.3 (4.565–6.545)	5.5 (4.5175–6.695)	6.1 (5.3–77.16)	8.941	0.011*
RBC (10^12^/L)	4.92 (4.6475–5.3975)	4.775 (3.69–5.185)	5.05 (4.56–5.46)	8.359	0.015*
PLT (10^9^/L)	259 (218.5–280.75)	226 (138–282.25)	283 (250–309)	17.942	<0.001*

	HC vs. MAFLD	HC vs. ALD	ALD vs. MAFLD
BMI (kg/cm^2^)	<0.001	<0.001	<0.001
TBIL (μmol/L)	0.536	0.004	<0.001
DBIL (μmol/L)	0.045	0.007	<0.001
ALT (IU/L)	<0.001	<0.001	0.16
AST (IU/L)	0.032	<0.001	<0.001
GGT (IU/L)	0.003	<0.001	<0.001
TP (g/L)	0.351	0.01	0.001
ALB (g/L)	0.723	0.002	<0.001
FPG (mmol/L)	0.039	<0.001	0.004
URIC (μmol/L)	<0.001	<0.001	0.040
TG (mmol/L)	<0.001	<0.001	0.184
CHOL (mmol/L)	0.007	0.062	0.997
HDL-C (mmol/L)	<0.001	<0.001	0.714
LDL-C (mmol/L)	0.002	0.01	0.911
WBC (10^9^/L)	0.004	0.846	0.026
RBC (10^12^/L)	0.941	0.01	0.012
PLT (10^9^/L)	0.003	0.043	<0.001

### 3.2 Fungal microbiome composition

**Figure 1:**
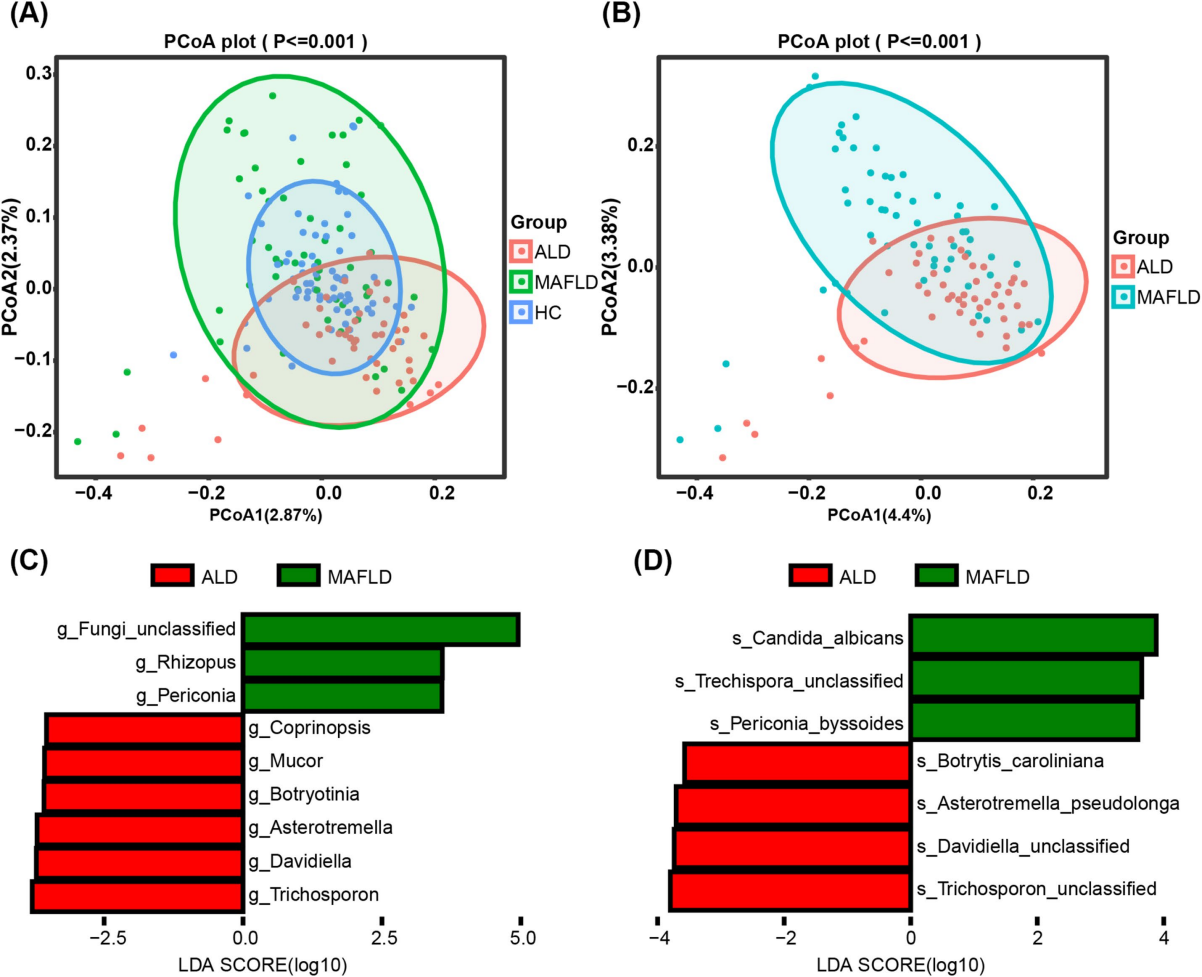
Significant differences in the gut fungal microbiomes of ALD and MAFLD. **(A)** Principal Coordinate Analysis (PCoA) of fungal biomes in ALD (*n* = 48), MAFLD (*n* = 55), and HC (*n* = 64). **(B)** PCoA of fungal biomes in ALD and MAFLD. **(C)** Linear discriminant analysis (LDA) of ALD genera with MAFLD. **(D)** Linear discriminant analysis (LDA) of ALD species with MAFLD. A *p* value of equal or less than 0.05 was considered as statistically significant.

Analysis of alpha diversity showed no significant differences across groups (Shannon Index, *p* = 0.95; Inverse Simpson Index, *p* = 0.95), as detailed in Supplementary Figures S1A,B. However, significant distinctions in community composition were observed using MANOVA based on the Jaccard index (*p* ≦ 0.001), illustrated in Figure 1A. Bray–Curtis dissimilarity further supported these findings. Notably, the fungal microbiome showed a significant contrast between the ALD and MAFLD groups according to the Jaccard index (*p* ≦ 0.001) (Figure 1B) and Bray–Curtis dissimilarity analysis further emphasized this disparity. Linear Discriminant Analysis (LDA) identified specific fungal taxa differentiating ALD from MAFLD. Notably, genera such as Trichosporon and Davidiella, and species like Trichosporon unclassified and Davidiella unclassified, were prevalent in ALD, while Rhizopus and *Candida albicans* (*C. albicans*) were more common in MAFLD (Figures 1C,D). Likewise, the relative abundance of the genus Periconia and species *C. albicans* was low in the ALD group compared with the MAFLD group (Supplementary Figures S2A,B). The relative abundances of the Mucor and Malassezia were significantly increased in the ALD cohort in relation to MAFLD (Supplementary Figures S2C,D).

### 3.3 Function annotation of fungal microbiota

**Figure 2:**
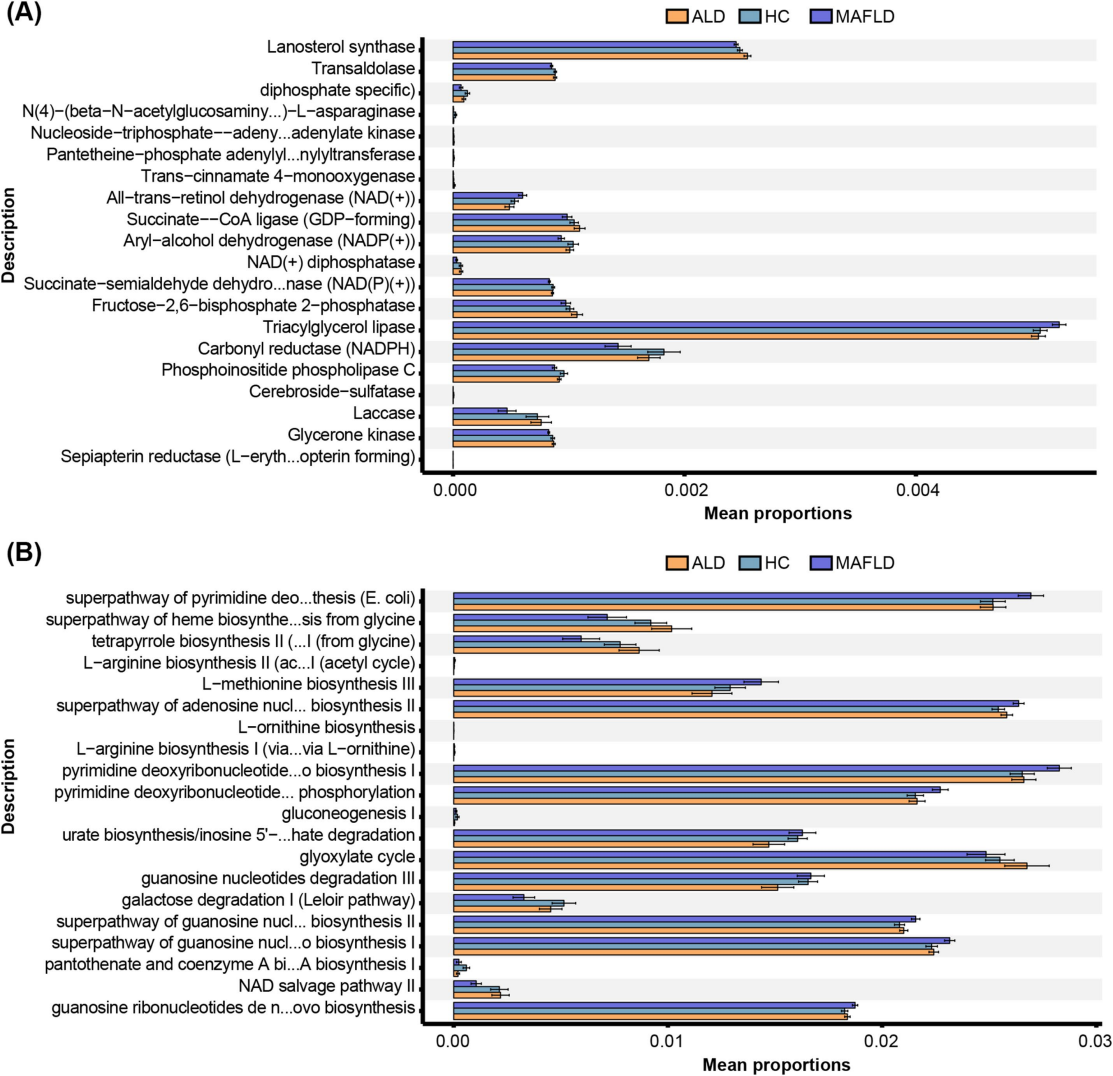
Function annotation of fungal microbiota. **(A)** Metabolic enzymes. **(B)** Metabolic pathways. Comparison of OTUs of function annotation in fungal microbiota among ALD, MAFLD, and HC patients. *N* = 167.

In ALD patients, significant reductions were noted in enzymes like aryl-alcohol dehydrogenase [NADP(+)], and carbonyl reductase (CBRs, NADPH) compared to HCs, with further decreases in MAFLD patients (Figure 2A). Metabolic pathways including the Leloir pathway and pantothenate and coenzyme A biosynthesis were downregulated in ALD compared to HCs, with more pronounced reductions in MAFLD (Figure 2B). Conversely, pathways related to adenosine nucleotide and pyrimidine biosynthesis were upregulated in both disease groups (Figure 2B).

### 3.4 A fungal signature differentiates ALD from MAFLD

**Figure 3:**
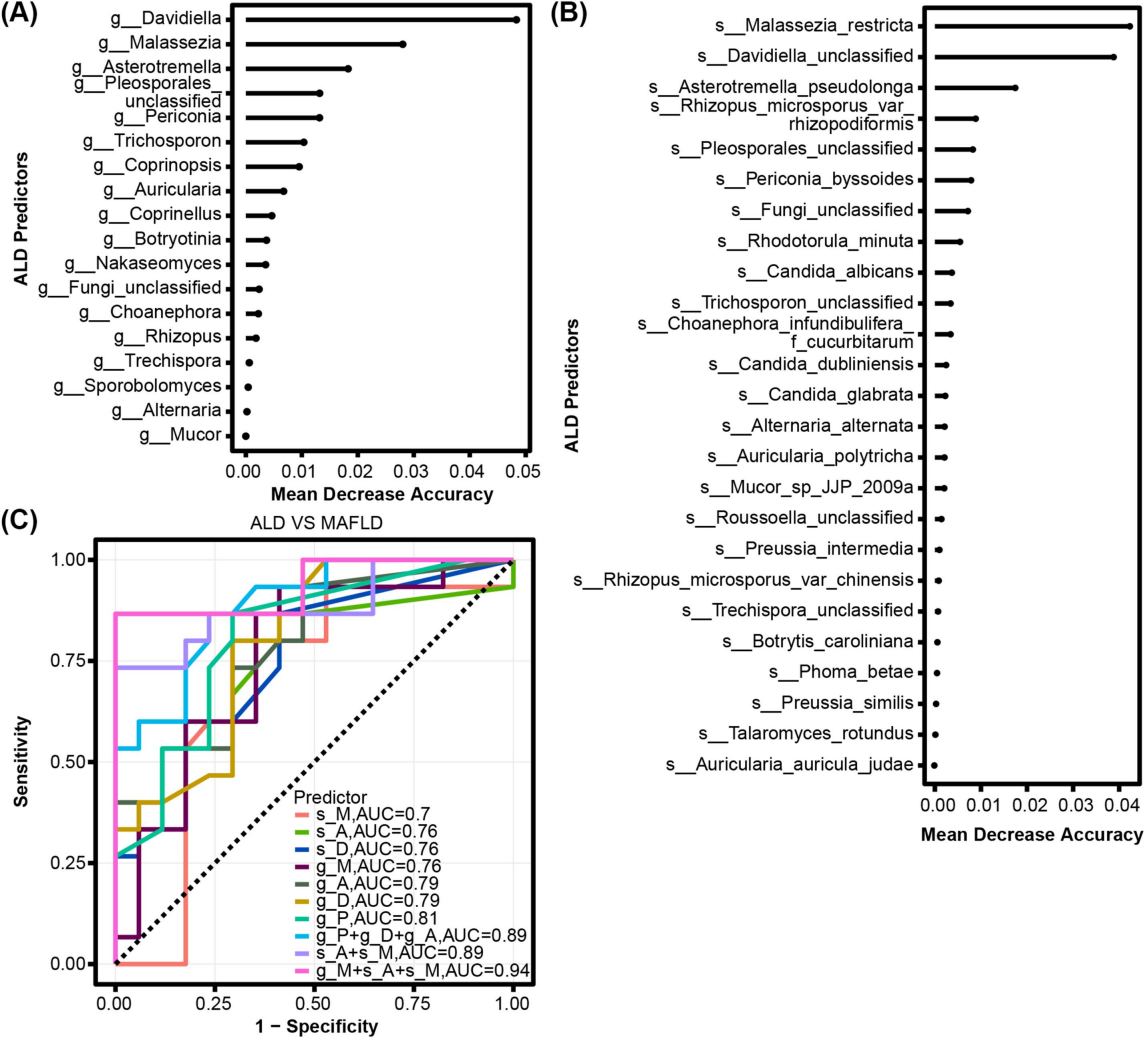
Fungal characteristics distinguish ALD from MAFLD. **(A)** Fungal genera and **(B)** species were quantified by Random Forest Analysis to determine the importance of their respective characteristics in detecting ALD and MAFLD. **(C)** ROC curves for fungal used to detect ALD vs. MAFLD.

We determined the mean decrease accuracy per random forest analysis to identify fungal genera and species with the highest feature importance for detecting ALD vs. MAFLD. The top genera distinguishing between ALD and MAFLD included Davidiella, Malassezia, Asterotremella, Pleosporales_unclassified, and Periconia (Figure 3A). The top species were Malassezia_restricta, Davidiella_unclassified and Asterotremella_pseudolonga (Figure 3B). In the second step, we determined genus, species, and combination discriminant values for ALD and MAFLD, respectively (Figure 3C). The highest area under the curve (AUC) value for a single fungal predictor was 0.81 for Pleosporales_unclassified, 0.79 for Davidiella and Asterotremella, 0.76 for Malassezia, Davidiella_unclassified, and Asterotremella_pseudolonga, 0.7 for Malassezia_restricta. We determined that the fungal signature consisting of species Asterotremella_pseudolonga, Malassezia_restricta and genus Malassezia had the highest discriminatory power with an AUC of 0.94 (Figure 3C and Supplementary Table S1). The sensitivity, specificity, accuracy, positive, and negative predictive value (PPV and NPV) for this fungal signature were 0.87, 1, 0.94, 1, and 0.89, respectively (Supplementary Table S1).

### 3.5 Subgroup analyses in MAFLD at various HS levels and body types

**Figure 4:**
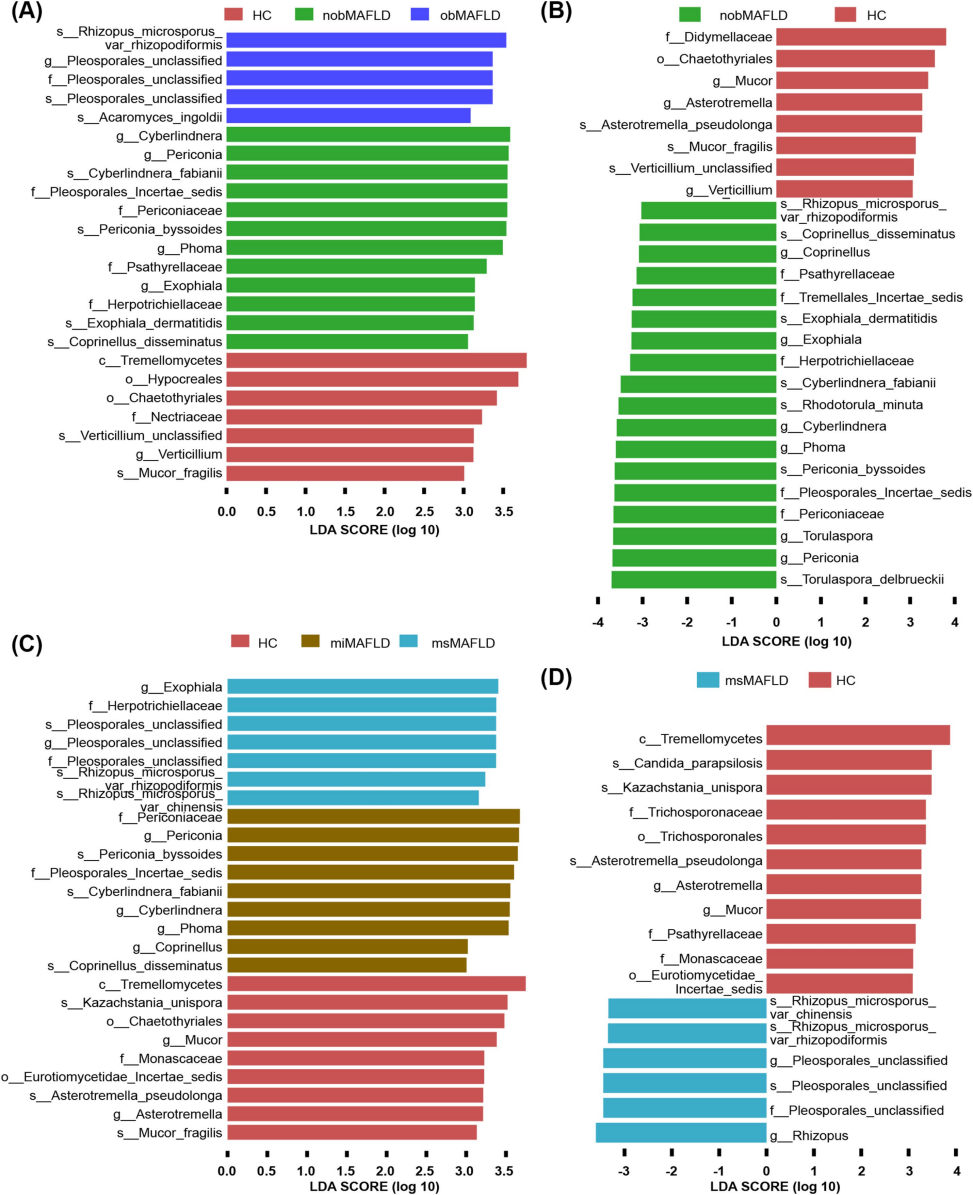
The most important fungal genera and species to identify MAFLD stratified by BMI or hepatic steatosis. **(A)** The comparison of fungal microbiota among nobMAFLD patients, obMAFLD patients and HC using the Lefse. **(B)** The comparison of fungal microbiota between nobMAFLD patients and HC using the Lefse. **(C)** The comparison of fungal microbiota among miMAFLD patients, msMAFLD patients and HC using the Lefse. **(D)** The comparison of fungal microbiota between msMAFLD patients and HC using the Lefse.

Periconia and Periconia_byssoides were predominantly found in non-obese MAFLD compared to obese MAFLD or HCs (Figures 4A,B). Similarly, Rhizopus_microsporus_var_chinensis, Rhizopus_microsporus_var_rhizopodiformis, and Pleosporales_unclassified showed elevated levels in MAFLD patients with moderate to severe HS relative to those with mild HS or HCs (Figures 4C,D).

### 3.6 Subgroup analyses in different progressions of ALD

**Figure 5:**
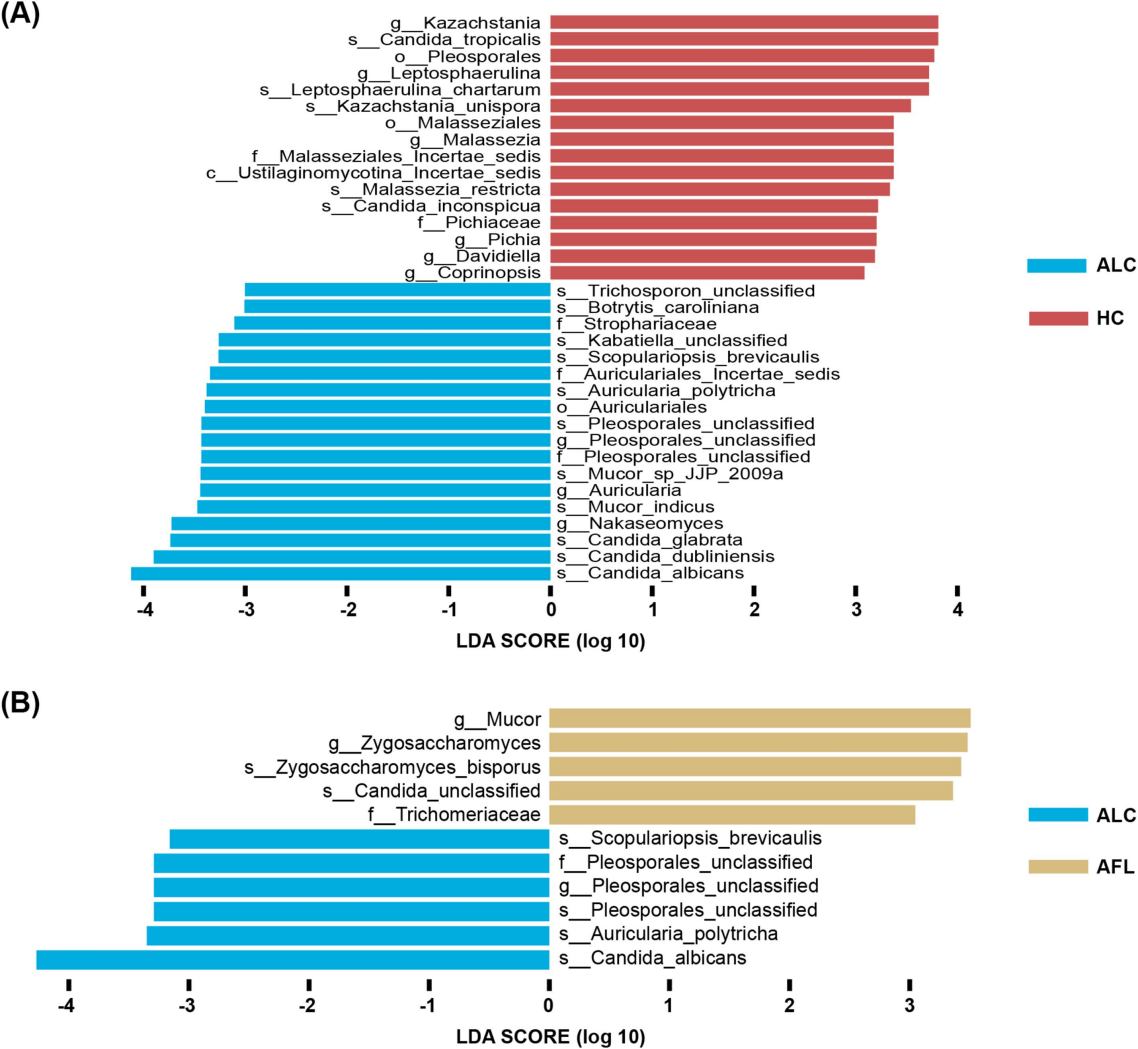
The most important fungal genera and species to identify ALD progresses. **(A)** The comparison of fungal microbiota between ALC patients and HC using the Lefse. **(B)** The comparison of fungal microbiota between ALC patients and AFL using the Lefse.

Distinct fungal profiles were associated with different stages of ALD. Genera like Pleosporales_unclassified and species including Candida_albicans, Auricularia_polytricha, Pleosporales_unclassified, and Scopulariopsis_brevicaulis were significantly elevated in patients with ALC compared to AFL or HCs (Figures 5A,B).

## 4 Discussion

The complexity of liver diseases such as ALD and MAFLD is exacerbated by their microbial interactions, notably through the fungal microbiome. This study provides novel insights into the differential fungal profiles between ALD and MAFLD, revealing that certain fungi not only differ between these conditions but also correlate with disease severity and progression. Of particular importance is the discovery of a distinct fungal profile, including Asterotremella_pseudolonga, Malassezia_restricta and Malassezia, capable of effectively distinguishing between ALD and MAFLD with high discriminatory power (AUC > 0.9).

Specifically in ALD patients, we observed an increase in Malassezia abundance compared to HCs and MAFLD, serving as a characteristic feature of ALD overall. For instance, Malassezia, traditionally known as a skin inhabitant for over a century (Grice and Dawson, 2017). Malassezia is classified into at least 14 species, 8 of which are isolated from human skin, including Malassezia restricta. Studies suggest that Malassezia can influence systemic metabolic pathways and immunological responses, potentially exacerbating liver disease conditions (Grice and Dawson, 2017; Prohic et al., 2016), emphasizing the need for further research into its systemic impacts. Recent research by Aykut et al. (2019) has demonstrated that Malassezia has the capability to migrate from the gut to the pancreas, potentially promoting pancreatic ductal adenocarcinoma by activating the mannose-binding lectin and complement-3 pathway. Additionally, colonization experiments with Malassezia have shown its ability to accelerate liver cancer progression (Zhang et al., 2023).

Moreover, the study highlights the role of *Candida albicans* in liver disease severity, particularly in ALD. The high abundance of *C. albicans* in more severe ALD cases suggests its potential as a biomarker for disease progression and severity. This finding aligns with existing research suggesting the pathogenic potential of *C. albicans* in various systemic diseases and its capability to affect host metabolic and immune responses (Aykut et al., 2019; Zhang et al., 2023; Demir et al., 2022). The opportunistic nature of *C. albicans* and its association with worsened disease outcomes necessitate a deeper exploration of its therapeutic targeting, possibly through antifungal treatments that could mitigate disease progression (Yang et al., 2017; Moyes et al., 2016). The oncogenic properties of *C. albicans* have been well-documented across various cancers (Vadovics et al., 2021). Specifically, elevated *C. albicans* levels can instigate immune dysregulation, synthesize tumor-promoting metabolites, activate pro-tumor signaling pathways, and upregulate prognostic marker genes associated with metastasis, collectively fostering tumor progression (Vadovics et al., 2021).

Beyond the increased abundance of *C. albicans*, Mucor species, typically opportunistic pathogens, have been identified as positively linked to hepatic inflammation and fibrosis (Demir et al., 2022). They are capable of causing mucormycosis, a systemic infection commonly seen in patients with hematological malignancies undergoing chemotherapy and those with uncontrolled diabetes (Petrikkos et al., 2012). Intriguingly, the presence of Mucor species in the fecal fungal microbiome of ALD patients distinguished MAFLD in our investigation. Apart from their involvement in mucormycosis onset, the significance of Mucor species in the commensal mycobiota remains ambiguous. *In vitro* studies have shown that Mucor administration can enhance intestinal permeability in epithelial cell monolayers (Mueller et al., 2019). Further investigations are warranted to elucidate the underlying factors contributing to the heightened abundance of Mucor species in ALD patients.

The influence of gut fungi in non-obese individuals with MAFLD, MAFLD subjects with severe HS, and the progression of ALD remains uncertain due to the limited sample size for subgroup analysis. Periconia is vital in MAFLD development in non-obese individuals in our study. Previous evidence has revealed that elevated Periconia levels may lead to mycotic keratitis (Gunasekaran et al., 2021). Notably, Pleosporales have the potential to predict the degree of HS in MAFLD and the progression of ALD, yet detailed mechanisms require further exploration.

Functional annotations related to metabolism, particularly CBRs, the Leloir pathway, and the biosynthesis of Pantothenic Acid and Coenzyme A, were dysregulated in liver disease patients relative to HCs in our study, representing a characteristic feature of fungal microbiota disorder in this population. CBRs, known as NADPH-dependent monomeric cytosolic enzymes with broad substrate specificity for various carbonyl compounds, play critical roles in diverse cellular and molecular processes involved in metabolism, detoxification, drug resistance, mutagenesis, and carcinogenesis of multiple drugs (Forrest and Gonzalez, 2000). The Leloir pathway, crucial for sugar metabolism, emerges as a promising therapeutic target for HCC (Tang et al., 2016). Pantothenic acid, a vital component of vitamin B5, serves as a key precursor for Coenzyme A biosynthesis, a fundamental cofactor essential for numerous metabolic reactions, including phospholipid synthesis, fatty acid metabolism, and the tricarboxylic acid cycle operation (Leonardi and Jackowski, 2007).

However, our study faces limitations, including the comparably less comprehensive fungal reference databases relative to bacterial databases, which may affect the accuracy of fungal community assessments. The observational nature of this study and the limited sample sizes necessitate cautious interpretation of the data and call for further longitudinal and interventional studies to validate these findings and explore the causative relationships between fungal dysbiosis and liver disease progression.

In conclusion, this study extends our understanding of the mycobiome’s role in liver diseases, revealing significant fungal taxa that differ between ALD and MAFLD. These findings pave the way for potential new biomarkers and therapeutic targets in managing chronic liver diseases.

## Data Availability

The ITS2 sequencing data presented in the study are deposited in the NCBI repository (https://www.ncbi.nlm.nih.gov/), accession number “PRJNA1172385”.
