# Supplementary material for: Different fungal signatures in ALD and MAFLD
Source: Front Microbiol. 2024 Nov 27;15:1510507. doi: 10.3389/fmicb.2024.1510507 (PMC11636606; doi:10.3389/fmicb.2024.1510507)
Supplement: Supplementary file 1 [file Table_1.DOCX]

***Supplementary Material***

1. **Healthy Control (HC) inclusion and exclusion criteria**

**Inclusion criteria**

1) Age: 18-65 years;

2) Healthy subjects without clinical signs or symptoms of disease, including evidence of viral hepatitis and metabolic syndrome, among others. (i) a healthy subject with no clinical signs or symptoms of disease, including evidence of viral hepatitis and metabolic syndrome

3) Imaging evidence of hepatic steatosis or cirrhosis can be excluded.

**Exclusion criteria**

1) Subjects who have not consumed alcohol in the past 12 months or have consumed less than 140 grams of ethanol per week (70 grams for women);

2) Those with an "abnormal" diet (e.g., vegetarian) in the past 12 months, or those who have been exposed to drugs such as antibiotics that disrupt the composition of the gut microbiome in the past 3 months.

1. **Supplementary Figures**


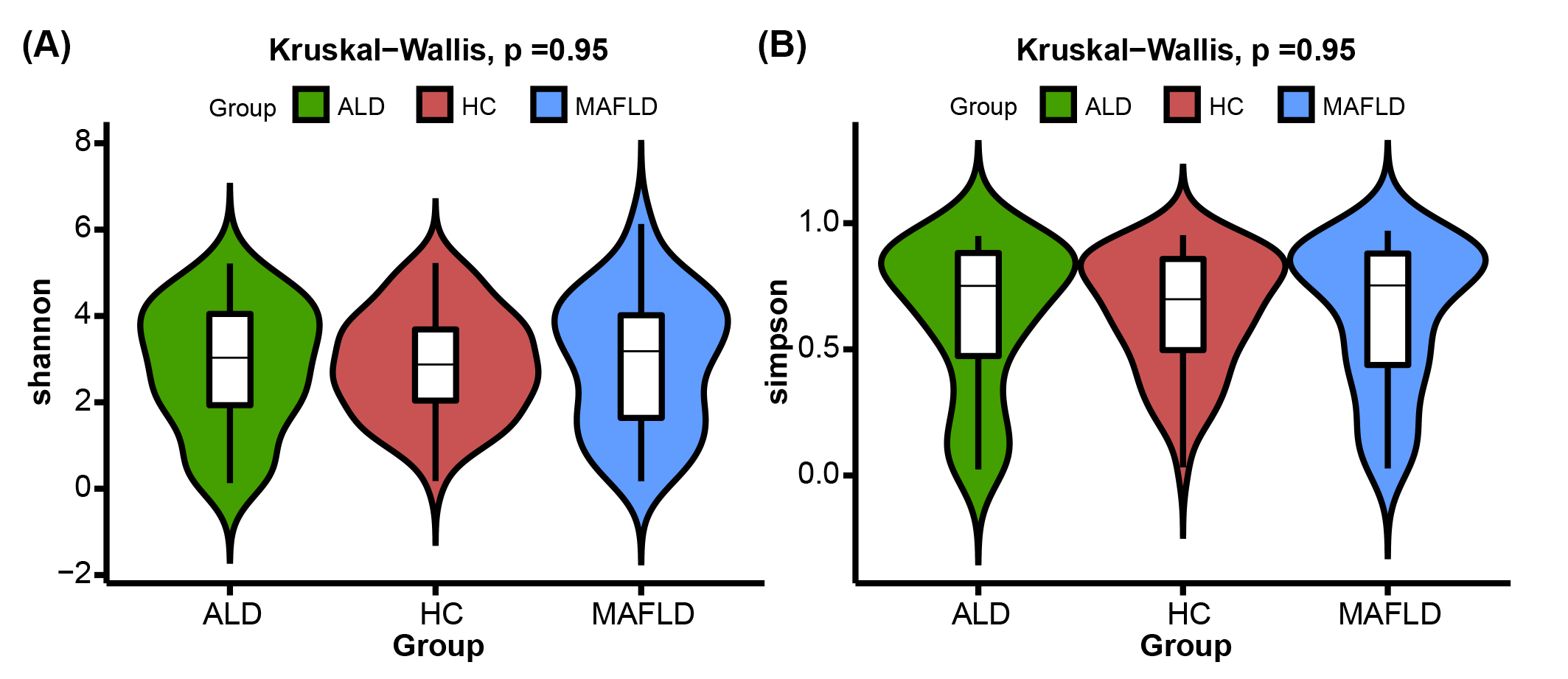
Supplementary Figure 1. Alpha diversity in ALD vs MAFLD vs HC. (A) Shannon index. (B) Inverse Simpson index. HC, n=64; ALD patients, n=48; MAFLD patients, n=55. ALD, alcohol-associated liver disease; HC, healthy controls; MAFLD, metabolic-associated fatty liver disease.


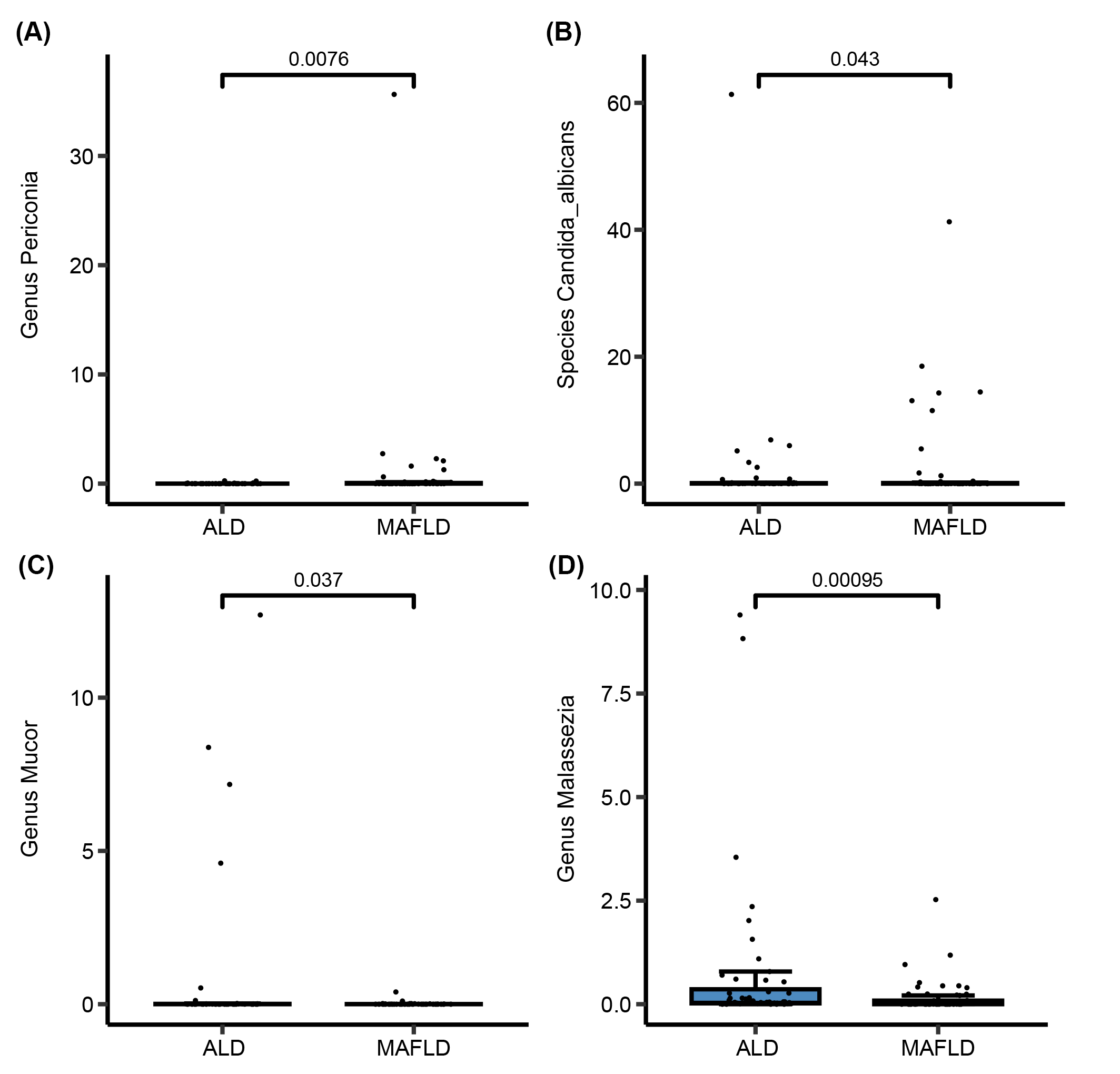


Supplementary Figure 2. Significant differences in the gut fungal microbiomes of ALD and MAFLD. (A) periconia (B) Candida albicans (C) Mucor, and (D) Malassezia. A p value of equal or less than 0.05 was considered as statistically significant.

1. **Supplementary Table 1**

Comparisons of fungal predictors between ALD and MAFLD patients.

| Feature | s__M | s__A | s__D | g__M | g__A | g__D | g__P | g__P+g__D+g__A | s__A+s__M | g__M+s__A+s__M |
| --- | --- | --- | --- | --- | --- | --- | --- | --- | --- | --- |
| AUC | 0.7 | 0.76 | 0.76 | 0.76 | 0.79 | 0.79 | 0.81 | 0.89 | 0.89 | 0.94 |
| Youden | 0.51 | 0.45 | 0.45 | 0.52 | 0.46 | 0.51 | 0.57 | 0.63 | 0.73 | 0.87 |
| Threshold | 0.18 | 0.05 | 0.07 | 0.02 | 0.03 | 0.34 | 0.05 | 0.5 | 0.55 | 0.42 |
| Sens | 0.8 | 0.87 | 0.87 | 0.93 | 0.93 | 0.8 | 0.87 | 0.87 | 0.73 | 0.87 |
| Spec | 0.71 | 0.59 | 0.59 | 0.59 | 0.53 | 0.71 | 0.71 | 0.76 | 1 | 1 |
| Acc | 0.75 | 0.72 | 0.72 | 0.75 | 0.72 | 0.75 | 0.78 | 0.81 | 0.88 | 0.94 |
| PPV | 0.71 | 0.65 | 0.65 | 0.67 | 0.64 | 0.71 | 0.72 | 0.76 | 1 | 1 |
| NPV | 0.8 | 0.83 | 0.83 | 0.91 | 0.9 | 0.8 | 0.86 | 0.87 | 0.81 | 0.89 |

s__M:s__Malassezia_restricta;s__A:s__Asterotremella_pseudolonga; s__D:s__Davidiella_unclassified; g__M:g__Malassezia；g__A：g__Asterotremella；g__D：g__Davidiella；g__P：g__Pleosporales_unclassified
